# Interactions and activities of factors involved in the late stages of human 18S rRNA maturation

**DOI:** 10.1080/15476286.2018.1564467

**Published:** 2019-01-13

**Authors:** Katherine Elizabeth Sloan, Andrew Alexander Knox, Graeme Raymond Wells, Claudia Schneider, Nicholas James Watkins

**Affiliations:** a Institute for Cell and Molecular Biosciences, The Medical School, Newcastle University, Newcastle upon Tyne, UK; b Department of Molecular Biology, University Medical Centre, Goettingen, Goettingen, Germany

**Keywords:** rRNA, nuclease, ribosome, methylation, RNA-protein interaction

## Abstract

Ribosome production is an essential cellular process involving a plethora of *trans*-acting factors, such as nucleases, methyltransferases, RNA helicases and kinases that catalyse key maturation steps. Precise temporal and spatial regulation of such enzymes is essential to ensure accurate and efficient subunit assembly. Here, we focus on the maturation of the 3ʹ end of the 18S rRNA in human cells. We reveal that human RIO2 is an active kinase that phosphorylates both itself and the rRNA methyltransferase DIM1 *in vitro*. In contrast to yeast, our data confirm that human DIM1 predominantly acts in the nucleus and we further demonstrate that the 21S pre-rRNA is the main target for DIM1-catalysed methylation. We show that the PIN domain of the endonuclease NOB1 is required for site 3 cleavage, while the zinc ribbon domain is essential for pre-40S recruitment. Furthermore, we also demonstrate that NOB1, PNO1 and DIM1 bind to a region of the pre-rRNA encompassing the 3ʹ end of 18S and the start of ITS1, *in vitro*. Interestingly, NOB1 is present in the cell at higher levels than other pre-40S factors. We provide evidence that NOB1 is multimeric within the cell and show that NOB1 multimerisation is lost when ribosome biogenesis is blocked. Taken together, our data indicate a dynamic interplay of key factors associated with the 3ʹ end of the 18S rRNA during human pre-40S biogenesis and highlight potential mechanisms by which this process can be regulated.

## Introduction

**Figure 1.**
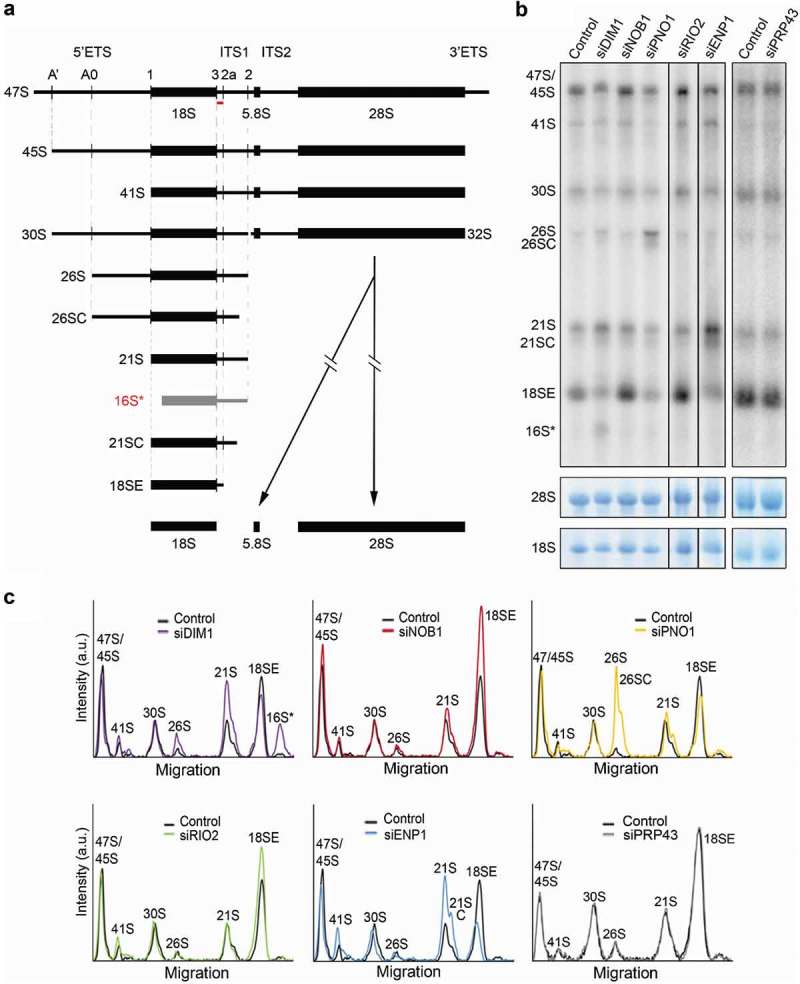
Ribosome biogenesis factors involved in the late stages of human 18S rRNA maturation. A) Schematic view of human small subunit pre-rRNA intermediates. Mature rRNAs are shown as black boxes, and internal and external transcribed spacers are shown as black lines (ITS – internal transcribed spacer; ETS – external transcribed spacer). Pre-rRNA cleavage sites are indicated by vertical lines on the initial 47S pre-rRNA transcript. The aberrant 16S* pre-rRNA species identified in DIM1 knockdown cells is shown in grey. The hybridisation position of the probe 5ʹETS1, used in panel B), is indicated by the red bar. B) HeLa cells were transfected with control siRNAs targeting firefly luciferase (Control) or siRNAs targeting the putative late pre-40S factors indicated. 60 h after siRNA transfection, RNA was extracted, separated by agarose-glyoxal gel electrophoresis and analysed by northern blotting using a probe hybridising to the 5ʹ end of ITS1 (A). As a loading control, mature 28S and 18S rRNA were detected by methylene blue staining. The identities of the pre-rRNA species detected are indicated to the side of the panels. C) The levels of pre-rRNA intermediates were normalised to the 28S rRNA and the relative intensity at each position was plotted relative to migration in the gel. The pre-rRNA species corresponding to each peak are indicated.

The assembly of eukaryotic ribosomes, which are composed of 4 ribosomal RNAs (rRNAs) and ~80 ribosomal proteins, is a highly complex and energy-consuming cellular process [1,2]. In the nucleolus, three of the four mature rRNAs, 18S, 5.8S and 28S (25S in yeast), are co-transcribed by RNA polymerase I as a single precursor (47S in humans) that also contains long external (ETS) and internal (ITS) transcribed spacers (Figure 1(A); [3,4]). The fourth rRNA, 5S, is transcribed by RNA polymerase III in the nucleoplasm from where it is imported into the nucleolus and integrated into the large ribosomal subunit (LSU) as part of the 5S RNP [2]. Maturation of the rRNAs involves a series of endonucleolytic cleavages and exonucleolytic processing to remove the ETS and ITS sequences, as well as the introduction of a myriad of rRNA modifications by small nucleolar RNPs (snoRNPs) and stand-alone enzymes to functionally important regions of the mature rRNA sequences [4–6]. An early pre-ribosomal complex (90S) is assembled onto the nascent pre-rRNA, which is then separated into precursors of the small subunit (SSU; pre-40S) and large subunit (pre-60S) [2]. These complexes undergo extensive maturation in the nucleolus prior to nucleoplasmic remodelling events that generate export-competent particles, which are transported to the cytoplasm where final maturation and quality control steps occur.

The assembly pathway of eukaryotic ribosomes is best-characterised in the yeast *Saccharomyces cerevisiae*, where more than 200 *trans*-acting biogenesis factors have been identified. The early stages of assembly and maturation of the SSU, which contains the 18S rRNA, are mediated by the SSU processome [2]. The later stages of SSU production are focused around the 3ʹ end maturation of the 18S rRNA. In yeast, this includes the *N*^6^,*N*^6^-dimethylation of adenosines 1781 and 1782 in the 18S rRNA by the methyltransferase Dim1 [7] and cleavage of the 3ʹ end of 18S rRNA at site D by the PIN-domain endonuclease Nob1 [8]. Interestingly, both Nob1 and Dim1 are recruited to early pre-ribosomal complexes but do not act until after pre-40S export to the cytoplasm [9], raising the question of how their catalytic activity is regulated. Crosslinking studies and structural analyses have revealed that Nob1 contacts two sites in the pre-rRNA, its cleavage site at the 3ʹ end of the 18S rRNA and helix 40 of the 18S rRNA, which is proposed to serve as a binding platform [10–12]. Furthermore, it has been suggested that conformational changes in late pre-40S complexes bring these sites in close proximity thereby regulating the timing of site D cleavage [10] and that the remodelling activity of the RNA helicase Prp43 (PRP43 or DHX15 in humans) contributes to Nob1 gaining access to its cleavage site [8,13]. The detection of overlapping binding sites for Nob1 and its partner Pno1 has led to a model in which Pno1 blocks site D, ensuring that endonucleolytic cleavage by Nob1 can only occur upon dissociation of Pno1 [11]. Since cleavage at the 3ʹ end of the 18S rRNA is one of the final events in SSU biogenesis that generates mature, translation-competent small ribosomal subunits, ensuring the correct assembly of the pre-40S complexes prior to Nob1-mediated cleavage is important. Consistent with this, the action of Nob1 is promoted by a proof-reading, translation-like cycle in which the large ribosomal subunit and the translation factor Fun12 (eIF5B) bind pre-40S complexes that have not been processed at site D [14,15]. Less is known about how the activity of Dim1 may be regulated but it has been shown that Pno1 is required for methylation of A1781 and A1782 by Dim1 [16]. Notably, late yeast pre-40S complexes contain two kinases of the Rio family, Rio1 and Rio2, which likely contribute to the regulation of cytoplasmic maturation steps [2]. However, while nucleotide binding by Rio1 is known to be necessary for Nob1-mediated cleavage of site D [11], no substrates of the kinase activity of these enzymes have been identified so far.

The whole process of ribosome biogenesis is considerably less well understood in humans. The fact that many ribosome biogenesis factors are conserved in eukaryotes suggests that the basic mechanisms of subunit assembly are similar. Indeed, the only key difference was originally thought to be the vertebrate-specific cleavage site in the 5ʹ ETS (A’). However, recent RNAi-based screens [17–19] and analyses of individual mammalian ribosome biogenesis factors (e.g. [20–24]) have uncovered additional human ribosome biogenesis factors and notable differences between the pathway of ribosome assembly in yeast and mammals. While the 3ʹ end of the yeast 18S rRNA is generated by a series of endonucleolytic cleavages, in humans, it has been shown that 3ʹ-5ʹ exonucleases, including the exosome, make a significant contribution to removal of ITS1 [18,23,25]. The contribution of exonucleases to 3ʹ maturation of the 18S rRNA in humans, which are not involved in yeast 18S rRNA 3ʹ processing, has recently been extended by the finding that the poly (A)-specific ribonuclease PARN performs the final trimming to generate the 18SE pre-rRNA that is exported to the cytoplasm [21,26]. However, as in yeast, the final 18S rRNA maturation step is an endonucleolytic cleavage of site 3 (analogous to yeast site D) by NOB1 [23,25,27,28]. Recently, hCINAP, which is homologous to the yeast cytoplasmic pre-40S NTPase Fap7, has been shown to be required for NOB1-mediated cleavage of site 3 *in vitro* [27]. Furthermore, the kinase domain of RIO2 is important for the recycling of NOB1 and other pre-40S biogenesis factors that shuttle been the nucleus and cytoplasm [29]. It has recently been shown that, as in yeast, the human homologue of Dim1 (DIM1/DIMT1L) catalyses the *N*^6^,*N*^6^-dimethylation of 18S-A1850 and 18S-A1851 in the loop of helix 45 [16]. Interestingly however, in contrast to yeast, where Dim1 is co-exported to the cytoplasm with pre-40S complexes, human DIM1 predominantly localises to the nucleolus and nucleoplasm where it has been suggested to act [16,30]. High resolution structures of late human pre-40S complexes have also recently been published [28]. In these structures PNO1 and NOB1 are found bound to the 3ʹ end of the 18S rRNA, with NOB1 held in an inactive conformation. However, it is currently unclear how NOB1 re-organises within the pre-40S structure to move its active site so that it can cleave site 3.

Defects in ribosome biogenesis underlie more than 20 genetic diseases, including Treacher Collins syndrome and Diamond Blackfan anaemia [31]. Perturbation of ribosome assembly affects the levels of the major tumour suppressor p53 (e.g. [32,33]) and changes in ribosome production have been linked to multiple forms of cancer. Indeed, the genes encoding several ribosomal proteins and ribosome biogenesis factors, including some of those involved in the late stages of pre-40S maturation, have been identified as ‘cancer genes’ [34]. Furthermore, recent work has indicated that changes in the late maturation stages of 18S rRNA processing occur during the diurnal change in liver mass in mice. Through an as yet uncharacterised pathway, conversion of the final 18S precursor to the mature 18S rRNA is impeded and the 18S precursor is polyadenylated and then degraded by the exosome [35]. Given the importance of ribosome production in human health and disease, detailed understanding of the human ribosome assembly pathway is required. As previous work from several labs has provided a relatively detailed view of the early, nucleolar aspects of human 18S rRNA maturation, here we focus on the later steps of pre-40S maturation. We further characterise the factors involved, exploring how the activities of key enzymatic proteins contribute to pre-40S maturation and how they are regulated.

## Results

### Characterisation of proteins involved in the late stages of human 18S rRNA maturation

While the factors involved in the final stages of pre-40S maturation in yeast are conserved in humans, several recent studies have highlighted important differences between the roles of ribosome biogenesis factors in yeast and humans (see for example [18–23],). To confirm the requirement for the human counterparts of several yeast proteins in 18S rRNA maturation in human cells and gain insight into the precise stages at which these proteins act, we used RNAi to deplete key factors predicted to be involved in the late stages of human 18S rRNA maturation and then examined the effects on pre-rRNA processing. siRNAs targeting the ribosome biogenesis factors DIM1 (DIMT1L), NOB1, PNO1 (DIM2), RIO2 (RIOK2), ENP1 (BYSL) and PRP43 (DHX15), or control siRNAs targeting firefly luciferase, were transfected into HEK293 cells and, 60 h later, RNA was extracted, separated by denaturing agarose gel electrophoresis and analysed by northern blotting using a probe hybridising to the 5ʹ end of ITS1 (Figure 1(A)). The efficiency of each knockdown was determined by western blotting using antibodies against the endogenous proteins, except in the case of DIM1, where due to the lack of a functional DIM1 antibody, we analysed the ability of the siRNAs to deplete FLAG-tagged human DIM1 stably expressed in HEK293 cells (Supplementary Figure S1).

Knockdown of either ENP1 or DIM1 resulted in reduced 18SE pre-rRNA levels and the accumulation of the 21S pre-rRNA (Figure 1(A–C)) consistent with previously published data [16,20,23]. Upon knockdown of ENP1, we also observed the accumulation of 21SC, indicating a defect in the exosome-mediated processing of the 21S pre-rRNA, as previously reported [20,23]. Depletion of DIM1 also resulted in an increase in 26S pre-rRNA levels and the accumulation of an aberrant pre-rRNA species, named 16S* (Figure 1(A–C)), as was previously noted [16]. Northern blotting, using a range of probes hybridising to different pre-rRNA regions, revealed that the aberrant pre-rRNA was detectable with ITS1 probes hybridising upstream of cleavage site 2 but not with a probe recognising the 3ʹ end of the 5ʹ ETS (Supplementary Figure S2A and B). Based on the sizes of the RNA species, this suggests that the 3ʹ end of 16S* is generated by site 2 cleavage, (Figure 1(A) and Supplementary Figure S2B). The detection of an aberrant, 5ʹ truncated form of the 21S pre-rRNA, which lacks the 5ʹ end of the 18S rRNA and is not part of the normal pre-rRNA processing pathway, implies that in cells lacking DIM1 pre-rRNAs are targeted for degradation. The knockdown of PNO1 resulted in reduced levels of the 18SE pre-rRNA, but also caused accumulation of the 26S pre-rRNA (as seen previously [18]), which extends from site A0 to site 2 and is rarely detected due to co-ordinated processing of the A0 and A1 sites. Notably, we also observed accumulation of a 3ʹ processed form of 26S that by analogy to 21SC, we termed 26SC (Figure 1(A–C)). Interestingly, detection of this intermediate implies that exosome-mediated trimming following site 2 cleavage can take place independent of cleavage at site A1. In contrast to the other knockdowns, depletion of NOB1 or RIO2 resulted in a reproducible accumulation of the 18SE pre-rRNA, indicating defects in the conversion of 18SE to mature 18S rRNA (Figure 1(A–C)), consistent with earlier work [21,23,29]. In contrast to our data, knockdown of NOB1 was also previously shown to cause the accumulation of the 26S pre-rRNA [21]. However, these knockdowns used different siRNAs and were performed for significantly longer. Finally, knockdown of the RNA helicase PRP43 had no significant effect on pre-rRNA processing, questioning whether this protein is required for the late stages of human pre-40S maturation (see discussion). Previous work has, however, shown the involvement of PRP43 in early stages of 5ʹ ETS processing [36]. While we cannot explain these differences between our data and that published previously, the previous analysis of PRP43 used different cells (HEK293 instead of HeLa) and different siRNAs. Furthermore, it is important to also note that in yeast depletion of Prp43 causes early, but not late defects in SSU production [37].

### PNO1, NOB1 and RIO2 are present in late 18S pre-rRNA processing complexes

**Figure 2.**
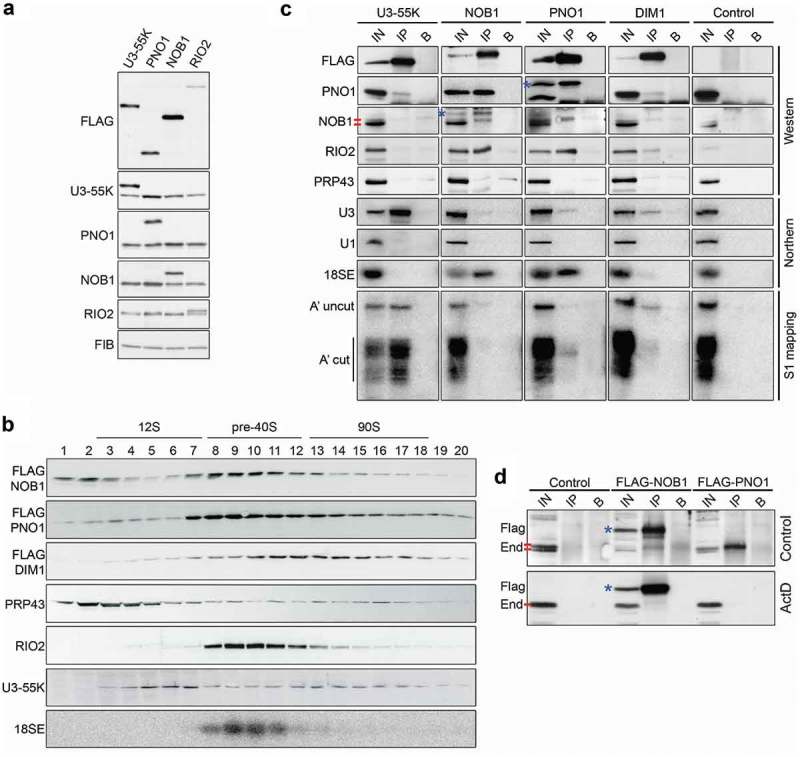
18S rRNA maturation factors associate and dissociate from pre-40S complexes at different times. A) HEK293 cells for expression of FLAG-tagged U3-55K, PNO1, NOB1 or RIO2 were treated with different concentrations of tetracycline to induce expression of FLAG-tagged versions of each protein to endogenous levels. Cells were harvested and proteins were analysed by SDS-PAGE followed by western blotting using the antibodies indicated to the left of the panel. The core box C/D snoRNP protein fibrillarin (FIB) served as a loading control. B) Expression of FLAG-tagged NOB1, PNO1 or DIM1 was induced to endogenous levels in HEK293 stable cell lines for 24 h before harvesting. Whole cell lysates were prepared and separated by glycerol gradient centrifugation. After fractionation, the protein content of each fraction was analysed by western blotting using an anti-FLAG antibody (NOB1, PNO1, DIM1) or antibodies against endogenous U3-55K, PRP43 or RIO2 (indicated to the left of each panel). Alternatively, RNAs in each fraction were extracted and analysed by northern blotting using a probe hybridising to the 5ʹ end of ITS1 to detect the 18SE pre-rRNA. Fraction numbers are given and the fractions containing 12S, pre-40S and 90S complexes (SSU processomes) are indicated. C) Expression of FLAG-tagged NOB1, PNO1 or DIM1, or the FLAG-tag alone (Control) was induced in HEK293 cell lines to the levels of the endogenous proteins. Whole cell extracts were prepared and used for immunoprecipitation experiments with protein G sepharose coupled to an anti-FLAG antibody or non-antibody-bound beads. Inputs (IN) and the eluates from anti-FLAG coupled beads (IP) or non-antibody-bound beads (B) were analysed by western or northern blotting using the antibodies or probes indicated to the left of the panel. * indicates FLAG-tagged NOB1 or PNO1, the red bars denote the two endogenous NOB1 bands. Alternatively, RNA eluates were subjected to S1 nuclease mapping using a radiolabelled probe hybridising across the A’ cleavage site in the 5ʹETS and the DNA fragments (A’ uncleaved; A’ cleaved) were detected using a phosphorimager. D) Control HEK293 cells or those expressing FLAG-tagged NOB1 or PNO1 were treated with 0.1 μg/μl actinomycin D (ActD) for 2 h or left untreated (control). Whole cell extracts were prepared and used in immunoprecipitation experiments with anti-FLAG-coupled or non-antibody-bound protein G sepharose. Inputs (IN; 10%) and the eluates from anti-FLAG coupled beads (IP) or non-antibody-bound beads (B) were analysed by western blotting using an antibody against endogenous NOB1. * indicates FLAG-NOB1, the red bars denote the two endogenous NOB1 bands (End), where present.

Having ascertained the requirement for these factors for the late stages of 18S rRNA processing using RNAi, we next set out to characterise the interactions that they form within human pre-ribosomal complexes. To achieve this, we generated HEK293 cells stably expressing tetracycline-inducible, FLAG-tagged PNO1, NOB1, RIO2 or DIM1 proteins. For comparison, we also used cells stably expressing a FLAG-tagged version of the U3 snoRNP protein U3-55K [38], which is known to act in early 90S pre-ribosomal complexes. In order to express the FLAG-tagged proteins at endogenous levels, we titrated the amount of tetracycline added so that the expression of each of the FLAG-tagged proteins was to a level equivalent to that of the endogenous protein, where protein-specific antibodies were available (Figure 2(A); lower panels). These expression conditions were used for all subsequent experiments except where indicated. Due to the lack of a functional antibody for DIM1, the expression level of FLAG-DIM1 was titrated to be similar to that of FLAG-U3-55K (Supplementary Figure S3), a core component of the SSU processome. Using these induction conditions, we determined the relative expression levels of these proteins by re-probing the membrane with anti-FLAG antibodies. Surprisingly, this revealed that while U3-55K and PNO1 are present at equal levels in the cell, NOB1 and RIO2 are present at significantly higher and lower levels respectively (Figure 2(A); upper panel).

We next used glycerol gradient centrifugation to analyse the co-migration of the FLAG-tagged proteins with pre-ribosomal complexes. Whole cell extracts prepared from the HEK293 cell lines expressing each of the FLAG-tagged proteins were separated by glycerol gradient centrifugation and the resultant fractions were analysed by both northern and western blotting. For comparison, and to provide markers for the early 18S rRNA processing complex, the SSU processome, we also analysed the migration of the U3 snoRNP via the U3-associated U3-55K protein in the gradients. The free U3 snoRNP, a 12S complex, was found in fractions 3–7, while the U3 snoRNP present in 90S complexes (SSU processome) was found in fractions 13–18 (Figure 2(B)). FLAG-tagged NOB1 was present as a free protein (fractions 1–3) and in pre-40S pre-ribosomal complexes containing the 18SE pre-rRNA (fractions 8–12). Only low levels of FLAG-NOB1 were found in the fractions containing the SSU processome. Using RIO2-specific antibodies, we detected endogenous RIO2 almost exclusively co-migrating with pre-40S complexes (fractions 8–12) with only low levels present in fractions containing the SSU processome. FLAG-tagged PNO1 co-migrated with both NOB1, RIO2 and 18SE in fractions 8–12 but in contrast to NOB1 and RIO2, a significant amount of PNO1 was also found in larger complexes (fractions 12–18) correlating with the SSU processome. FLAG-tagged DIM1 was observed to primarily co-migrate with the large, SSU processome complexes (fractions 12–18) and was only detected at low levels in fractions containing the 18SE pre-rRNA and late pre-40S complexes. Our data are therefore consistent with earlier work showing the presence of NOB1, PNO1 and RIO2 in later pre-40S complexes [28–30,39,40]. Consistent with the mild early pre-rRNA processing defects [36] and lack of late pre-rRNA processing defects (Figure 1(B–C)) observed upon PRP43 depletion, we only observed low levels of PRP43 in fractions containing pre-ribosomal complexes. PRP43 was previously shown to be associated with early pre-ribosomal particles [36]. We did not observe this in our experiments. This could be due to the difference in gradient conditions as the earlier work used sucrose, instead of glycerol, and a lower salt concentration (100 mM KCl instead of 150 mM NaCl) in the buffer.

The glycerol gradient data suggest that the proteins analysed are recruited to pre-ribosomal complexes at different times (early, SSU processome-containing complexes and late, 18SE-containing pre-ribosomes) and remain associated for variable durations. To further clarify this, we next used immunoprecipitation experiments to confirm the association of these proteins with distinct pre-ribosomal complexes in the cell (Figure 2(C)). Whole cell extracts were prepared from HEK293 cells stably expressing the FLAG-tagged NOB1, PNO1 and DIM1 proteins. As controls, we also used extracts from cells stably expressing FLAG-U3-55K to verify the efficient isolation of early nucleolar pre-ribosomal particles or extracts from cells expressing the FLAG-tag alone (Control). Bait and associated proteins were analysed by western blotting using antibodies against the FLAG tag, PNO1, NOB1, RIO2 and PRP43, while co-precipitated RNAs were identified by northern blotting using probes against the U3 snoRNA, the 18SE pre-rRNA and the U1 small nuclear RNA (snRNA) as a negative control. In addition, the isolated RNAs were subjected to an S1 nuclease assay [41], using a 5ʹ-labelled probe that spans the A’ cleavage site in the 5ʹETS to determine the relative amounts of A’ uncleaved (47S) and A’ cleaved (45S and 30S) pre-rRNA transcripts associated with each protein.

As expected, none of the analysed pre-40S proteins or pre-rRNAs were co-precipitated by the FLAG tag alone, and the U1 snRNA was not present in the eluates of any of the immunoprecipitation experiments. Also, in line with its presence in the U3 snoRNP, FLAG-U3-55K associated robustly with the U3 snoRNA and both A’ cleaved and non-cleaved 5ʹETS-containing pre-rRNAs, but not the late 18SE pre-rRNA (Figure 2(C)). Interestingly, low levels of PNO1, RIO2, 18SE and the U3 snoRNA, but only background levels of NOB1, co-purified with FLAG-DIM1 (Figure 2(C)). These interactions are supported by the detection of A’ uncleaved and cleaved 5ʹETS pre-rRNAs in the FLAG-DIM1 eluates and are consistent with the gradient data suggesting that DIM1 is associated primarily with large, early pre-40S complexes (Figure 2(B)). This is in line with the recent finding that, in contrast to yeast Dim1, which is co-exported to the cytoplasm with pre-40S particles, in human cells DIM1 predominantly localises in the nucleolus and nucleoplasm [16,30]. FLAG-PNO1 also co-purified low levels of the U3 snoRNA and 5ʹETS-containing pre-rRNAs, implying that PNO1 is recruited to early pre-ribosomal particles. In addition, PNO1 was retrieved in the FLAG-U3-55K immunoprecipitation experiment further supporting this model. However, consistent with the gradient data suggesting that PNO1 is present in both the early and late pre-40S complexes (Figure 2(B)), FLAG-tagged PNO1 also co-purified the 18SE pre-rRNA, RIO2 and NOB1 (Figure 2(C)).

FLAG-tagged NOB1 co-purified PNO1, RIO2, the 18SE pre-rRNA and surprisingly, endogenous NOB1 (Figure 2(C,D)). It is interesting to note that in some experiments two forms of NOB1 were observed and that it was always the upper NOB1 band that co-purified with the late pre-40S processing factors, including FLAG-tagged NOB1. However, the two forms were not consistently observed (compare panels A and C in Figure 2) and therefore we cannot make any statements about what could represent a post-translational modification. NOB1 was, however, not associated with the U3 snoRNA, or the 5ʹ ETS-containing pre-rRNAs, supporting our prior observations suggesting that NOB1 is present in late, pre-40S complexes but not early, SSU processome complexes (Figure 1(B,C), Figure 2(B)). This is in line with earlier purification and localisation experiments [28,29,39,40]. Consistent with the lack of defects in the late stages of SSU pre-rRNA processing upon PRP43 depletion, we did not find PRP43 stably associated with late pre-40S complexes, as indicated by the lack of association with PNO1 or NOB1, implying that this protein may not be involved in the late stages of 18S rRNA maturation in humans (see discussion). However, we cannot rule out the possibility that this protein has a redundant function in the late stages of ribosome biogenesis in human cells. Taken together, our data indicate that DIM1 is present in early pre-40S complexes and PNO1 is present in both early and late complexes, while NOB1 and RIO2 are enriched in late pre-40S complexes.

To further dissect the timing of the interaction between NOB1 and PNO1, and better understand the production and interactions of the two alternative forms of NOB1, we next analysed the effects of impairing ribosome biogenesis on these interactions. To perturb ribosome biogenesis, we used low levels of actinomycin D (ActD), which blocks RNA polymerase I transcription. Immunoprecipitation experiments were performed using extracts from cells expressing FLAG-tagged NOB1 or PNO1 and the association with NOB1 was determined by western blotting. As we observed earlier (Figure 2(C)), both FLAG-PNO1 and FLAG-NOB1 specifically co-precipitated NOB1 (Figure 2(D); upper panel). Furthermore, the association of either FLAG-PNO1 or FLAG-NOB1 with endogenous NOB1 was lost after treatment of the cells with ActD (Figure 2(D)), indicating that these proteins only interact when they participate in ribosome biogenesis. These data imply that ongoing ribosome biogenesis is required for NOB1 multimerisation and also for the interaction between NOB1 and PNO1 in the cell.

### DIM1 dimethylates the 21S pre-rRNA in the nucleus

**Figure 3.**
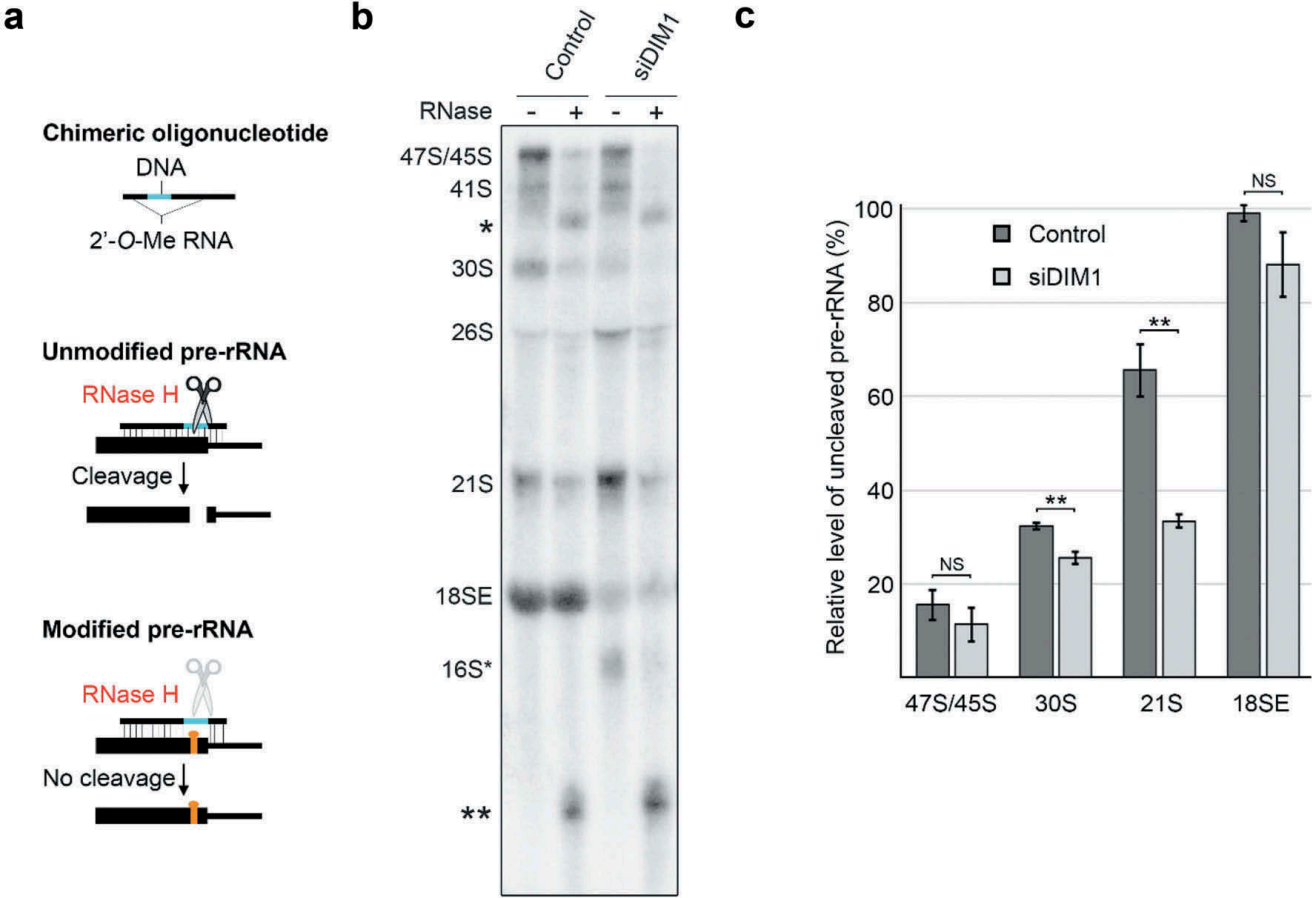
DIM1 primarily dimethylates the 21S pre-rRNA in humans. A) Schematic view of the RNase H-based assay for the detection of rRNA modifications. B) Total RNA was isolated from HeLa cells that had been transfected with control siRNAs or siRNAs against DIM1. Equal amounts of RNA (4 µg) were annealed to a chimeric RNA-DNA oligonucleotide that directs RNase H cleavage at 18S-A1850/1 and were either treated with RNase H (+) or left untreated (-). RNAs were re-isolated, separated by agarose-glyoxal gel electrophoresis and analysed by northern blotting using a probe that hybridises to the 5ʹ end of ITS1. The pre-rRNA species and cleavage products (* and **; see Supplemental Figure S4) are indicated on the left. C) The levels of the 47S/45S, 30S, 21S and 18SE pre-rRNAs detected in untreated and RNase H-treated RNA from cells treated with control siRNAs or those targeting DIM1 were quantified, using Image Quant software, from three independent experiments. For each precursor, the percentage uncleaved (i.e. representing DIM1 methylated pre-rRNA), compared to the equivalent RNA species that were not treated with RNase H, was plotted as mean ± standard error. **p < 0.01, t-test, NS – not significant.

The role of human DIM1 in catalysing the evolutionarily conserved 18S-m^6^_2_A1850 and 18S-m^6^_2_A1851 modifications has recently been confirmed [16]. While these modifications are introduced in the cytoplasm in yeast, human DIM1 has been shown to localise predominantly to the nucleolus [30] and primer extension analyses on human pre-rRNA have suggested that DIM1 methylation can take place before generation of the 18SE precursor [16]. This is supported by our finding that DIM1 is not present in late, pre-40S complexes with proteins such as NOB1. It remains unclear, however, which pre-rRNA species can be methylated by DIM1 and to what extent the sites are modified. To address this, we utilised a site-specific RNase H-based cleavage assay to detect changes in the modification status of 18S rRNA precursors upon RNAi-mediated depletion of DIM1 (Figure 3(A)). Dimethylation of the 18S rRNA sequence by DIM1 impedes the base-pairing capacity of the two target adenines and prevents annealing with the DNA component of a chimeric 2ʹ-*O*-methylated RNA-DNA oligonucleotide, therefore blocking RNase H-mediated cleavage at the 18S rRNA A1850/1 methylation sites. RNA was extracted from cells that had been transfected with a control siRNA or cells in which DIM1 levels were reduced by RNAi treatment. Equal amounts of RNA from each of the siRNA-treated cells were annealed to the chimeric oligonucleotide and half the sample was treated with RNase H while the other half remained untreated. Both the cleaved and uncleaved RNAs were separated by denaturing agarose gel electrophoresis and analysed by northern blotting using a probe specific to the 5ʹ end of ITS1 (Figure 3(B); * for 47S/45S and ** for 30S/21S-derived cleaved pre-rRNAs). A schematic representation of the expected pre-rRNA fragments generated by RNase H cleavage of unmodified 18S rRNA are shown in Supplementary Figure S4. Analysis of the pattern of detected fragments confirmed specific cleavage by RNase H at the intended target site (18S-m^6^_2_A1850/18S-m^6^_2_A1851).

The 18SE pre-rRNA derived from control cells was protected from cleavage by RNase H (Figure 3(C)) demonstrating that these positions are fully modified *in vivo*. In contrast, ~15% of the 47S/45S pre-rRNA was protected from cleaved by RNase H, indicating little or no modification of these early precursors. Interestingly, about 33% and 65% of the 30S and 21S pre-rRNAs were protected from RNase H cleavage, respectively. This demonstrates that DIM1-mediated dimethylation begins on the 30S pre-rRNA, and that a significant proportion of transcripts are dimethylated at positions 1850 and 1851 when the 21S pre-rRNA is produced. Knockdown of DIM1 resulted in a statistically significant reduction in the methylation of 18S-A1850/1 on both the 30S and 21S pre-rRNAs. This was seen through a decrease in the proportion of the uncleaved 30S pre-rRNA (from 33% to 25%) and 21S pre-rRNA (from 65% to 33%) in DIM1 knockdown cells as compared to control cells. Depletion of DIM1 leads to a significant decrease in the levels of the 18SE pre-rRNA (Figure 1(B,C)) and it is interesting to note that the small amount of the 18SE pre-rRNA that is produced in the DIM1 knockdown cells is almost completely modified at positions A1850/1. It is likely that this reflects pre-rRNAs that were bound by residual DIM1 in the knockdown cells and that were therefore modified and processed to form the 18SE precursor. Our data indicate that DIM1-mediated methylation of the 18S rRNA sequence begins on the 30S pre-rRNA and that once the 21S precursor is produced, the majority of the transcripts are methylated.

### Identification of protein-protein interactions in late pre-40S complexes

**Figure 4.**
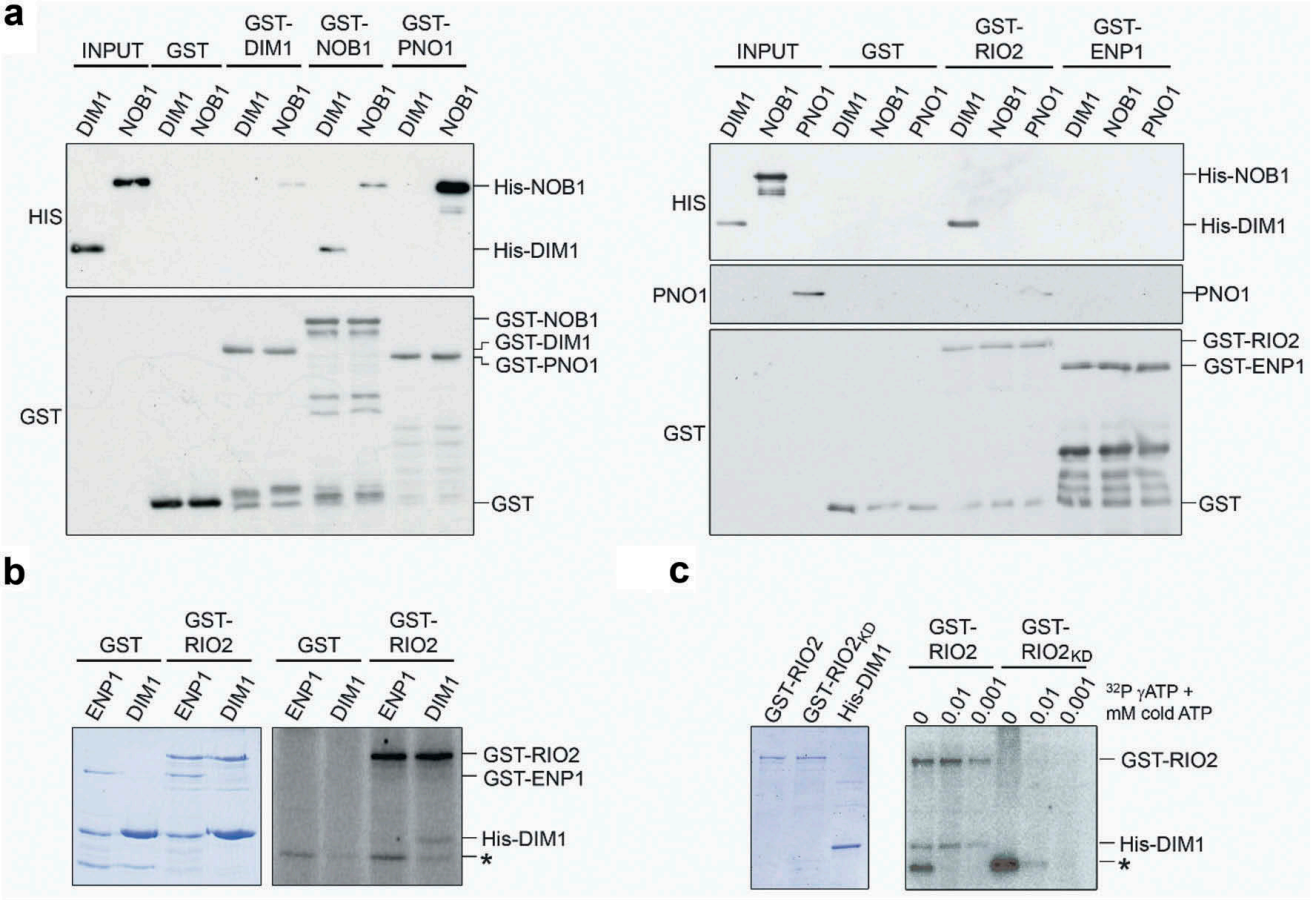
Identification of direct interactions between late-acting 18S maturation factors. A) Recombinant GST-tagged DIM1, NOB1, PNO1, RIO2 or ENP1, or the GST-tag alone, were incubated with His-tagged DIM1 or NOB1, or untagged PNO1 and complexes formed were retrieved on glutathione sepharose. Protein inputs (10%) and eluates were separated by SDS-PAGE, and bait and co-precipitated proteins were detected by western blotting using the antibodies indicated to the left of each panel. The bands corresponding to each full length recombinant protein are indicated to the right of each panel. B) His-DIM1 or GST-ENP1 were incubated with GST-RIO2 or GST in the presence of γ[^32^P]-labelled ATP and 0.001 mM cold ATP. C) His-DIM1 was incubated with either GST-RIO2, or GST-RIO2_KD_, with γ[^32^P]-labelled ATP and varying concentrations of cold ATP (indicated above each lane). Proteins in panels (B) and (C) were separated by SDS-PAGE, alongside non-labelled protein inputs, and detected by Coomassie staining or using a phosphorimager, respectively. The bands corresponding to the input proteins are marked on the right and a non-specific phosphorylated protein (~30 kDa) is indicated by an asterisk.

Having defined the pre-40S complexes with which these late-acting SSU biogenesis factors associate, to better understand the interplay between these factors and their roles in pre-40S maturation, we next determined whether they form direct interactions with each other. To do this, DIM1, NOB1, PNO1, RIO2 and ENP1 were recombinantly expressed with either a GST- or His-tag in *E. coli* and purified. To enable putative interactions between PNO1, RIO2 and ENP1 to be investigated, we generated an untagged form of PNO1 by protease cleavage to remove the GST-tag from the GST-tagged protein. To test for protein-protein interactions, GST-tagged proteins were incubated with His-tagged/untagged proteins and the complexes formed were then isolated using glutathione sepharose. The retrieved proteins were then analysed by western blotting (Figure 4(A)).

As anticipated from previous work in yeast [42], and also a recent paper analysing the human proteins [43], a significant amount of His-NOB1 co-purified with GST-PNO1, indicating a robust interaction between these proteins also in human cells. His-DIM1 was also efficiently co-precipitated with GST-RIO2, revealing a significant interaction between these two proteins. Furthermore, we detected reciprocal interactions between NOB1 and DIM1 (GST-NOB1/His-DIM1 and GST-DIM1/His-NOB1), and between GST-RIO2 and PNO1, but the relatively low amounts of prey proteins retrieved suggest that these interactions are relatively weak. Interestingly, we found that low levels of His-NOB1 co-purified with GST-NOB1, indicating that this protein has the capacity to dimerise/multimerise, as was originally proposed in yeast [44] and as observed in human cells (Figure 2(C,D)). No interactions were detected between any of the proteins tested and either GST alone or GST-ENP1, suggesting that the observed interactions are specific and that while ENP1 is associated with late pre-40S complexes, it does not directly contact other late-acting biogenesis factors. We have therefore uncovered direct protein-protein interactions between NOB1 and both PNO1 and DIM1, and also for RIO2 with both DIM1 and PNO1, that are likely important for human 40S subunit maturation. Interestingly, apart from the PNO1-NOB1 interactions, none of the other interactions would be predicted from the available human pre-40S structures [28].

### RIO2 is an active kinase that phosphorylates DIM1 in vitro

Our identification of DIM1 as a robust interaction partner of the putative kinase RIO2 raised the possibility that DIM1 may be phosphorylated by RIO2. This has been previously proposed for yeast Dim1 and Rio2, but not yet demonstrated [42]. Both archaeal and *Chaetomium thermophilum* (*Ct*) Rio2 have been shown to undergo autophosphorylation but so far, no other substrates have been identified for this kinase [29,45,46]. Indeed, based on the crystal structure of *Ct* Rio2, it has been suggested that Rio2 is an atypical kinase that does not phosphorylate other proteins during ribosome biogenesis [45]. To test whether human RIO2 is an active kinase and if DIM1 represents a substrate, we incubated recombinant GST-RIO2, or GST alone, with recombinant DIM1 or, as a control, ENP1 in the presence of [^32^P]-γ-ATP and 0.001 mM cold ATP. The reaction mixtures were separated by SDS-PAGE and then analysed by Coomassie staining to verify protein loading and using a phosphorimager to detect [^32^P]-labelled proteins. In the sample containing both RIO2 and DIM1, but not GST and DIM1, radiolabelled bands corresponding to both GST-RIO2 (approx. 100 kDa) and His-DIM1 (approx. 40 kDa) were detected (Figure 4(B)), suggesting that human RIO2 can phosphorylate itself and that DIM1 is also a substrate of its kinase activity. In addition, a labelled protein of ~30 kDa that did not correlate with any of the major proteins used was detected in both the samples containing GST-RIO2 and GST alone, suggesting that phosphorylation of this protein is not mediated by GST-RIO2 but instead is likely due to a contaminating *E. coli* kinase co-purified on glutathione sepharose. We next extended our assay to verify the specificity of the phosphorylation reactions. Incubation of GST-RIO2 with NOB1, ENP1 or PNO1, a protein we also identified as a direct interaction partner of human RIO2 (Figure 4(A)) did not lead to phosphorylation of any of these substrates, while the detection of RIO2 autophosphorylation confirmed the presence of active RIO2 kinase in these samples (Supplementary Figure S5A).

To demonstrate that DIM1 phosphorylation was mediated by RIO2, we introduced point mutations to disrupt the kinase domain of RIO2 (K123A and D246A; RIO2_KD_), as previously described [29]. At the ATP concentrations used in Figure 4(B) (0.001 mM cold ATP), phosphorylation of both RIO2 and DIM1 was observed in the presence of wild-type RIO2, but not when the RIO2_KD_ mutant protein was used (Figure 4(C) and Supplemental Figure S5B). The *Ct* RIO2 was previously shown to be more active at autophosphorylation at low concentrations of ATP [45]. We therefore tested phosphorylation of RIO2 and DIM1 in the presence of 0.01 mM cold ATP or the absence of cold ATP. The decrease in the concentration of cold ATP led to more efficient labelling of both RIO2 and DIM1. Interestingly, the 30 kDa labelled band became more prominent at lower ATP concentrations and its phosphorylation was stronger in the absence of RIO2 catalytic activity. In contrast, even at these low ATP concentrations, the RIO2_KD_ mutant protein showed no sign of (auto)phosphorylation activity. Our data therefore confirm that human RIO2 can phosphorylate both itself and DIM1.

### NOB1 and PNO1 bind cooperatively to helix 40 of the 18S rRNA in vitro

**Figure 5.**
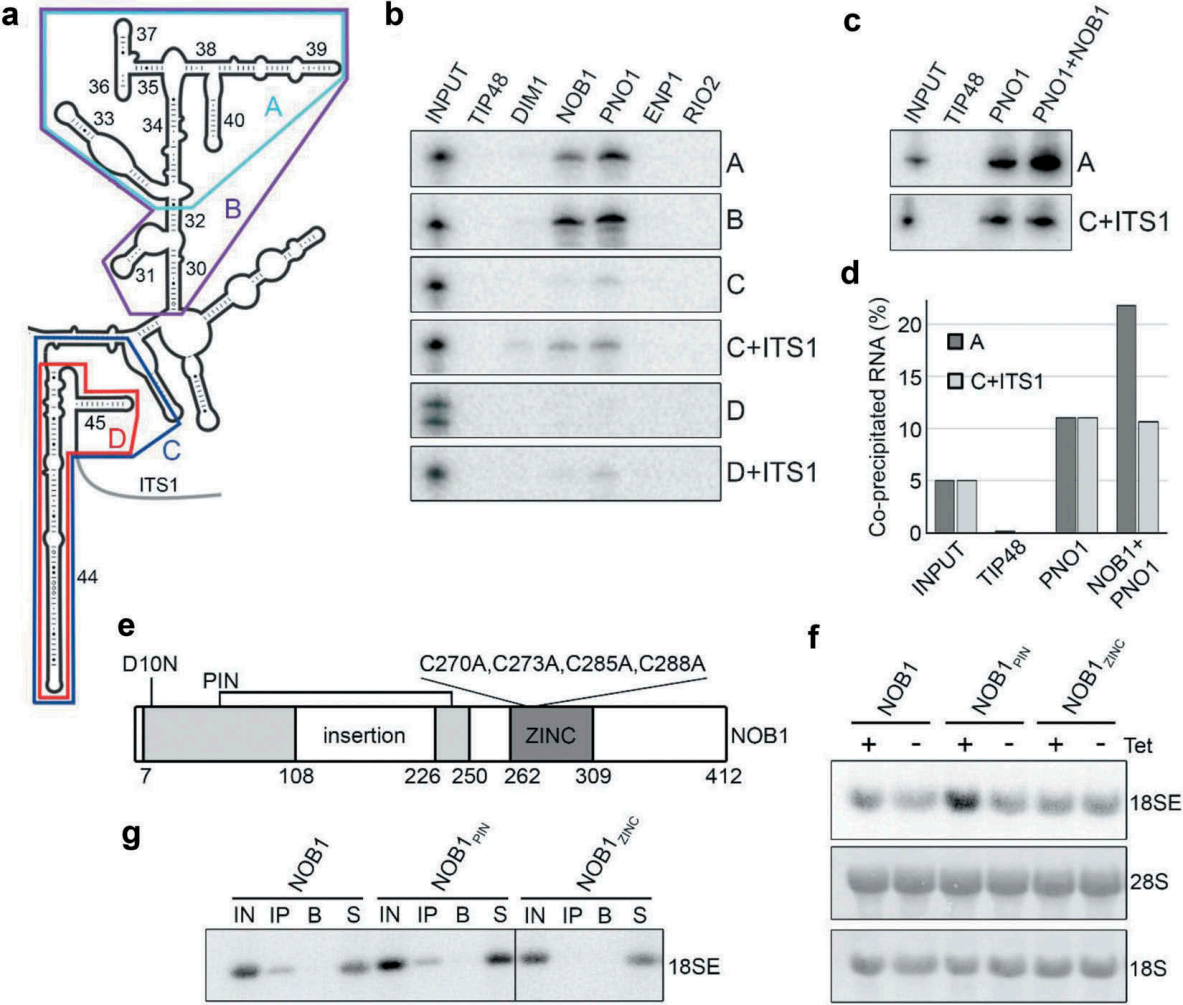
NOB1 and PNO1 bind two regions of the pre-rRNA *in vitro* and the zinc ribbon domain of NOB1 is important for integration into the pre-ribosome *in vivo*. A) Secondary structure of the 3ʹ major and minor domains of the 18S rRNA is shown with the positions of fragments used for protein-RNA binding analysis highlighted in colours. B) The purified GST-tagged proteins indicated were immobilised on glutathione sepharose and [^32^P]-labelled *in vitro* transcribed (pre-) rRNA fragments (shown in A) were added. After washing steps, co-purified RNAs were separated by denaturing polyacrylamide gel electrophoresis alongside RNA inputs (5%) and visualised using a phosphorimager. C) Protein-RNA interaction analyses were performed as in (B) using GST-PNO1 or a pre-formed complex of GST-PNO1 and His-NOB1. D) The relative amount of transcript co-precipitated in each sample in panel E), was quantified and is shown compared to the input sample. E) Schematic view of the structural domains of the nuclease NOB1 (adapted from [12,28]). Amino acid numbers corresponding to the domain boundaries and mutated residues are indicated. D10N – NOB1_PIN_; C270A, C273A, C285A and C288A – NOB1_ZINC_. F) Over-expression of FLAG-tagged full-length wild type NOB1, NOB1_PIN_ or NOB1_ZINC_ was induced in HEK293 stable cell lines for 24 h. Total RNA was then extracted and analysed by northern blotting using a probe hybridising to the 5ʹ end of ITS1 to detect the 18SE pre-rRNA. Mature 28S and 18S rRNA were visualised by methylene blue staining. G) Extracts prepared from HEK293 cells expressing FLAG-tagged wild type NOB1, NOB1_PIN_ or NOB1_ZINC_ were used in immunoprecipitation experiments with anti-FLAG-coupled (IP) or non-antibody-bound (B) protein G sepharose. Inputs (IN; 10%), eluates (IP and B) and non-precipitated RNAs (S) were separated by agarose-glyoxal gel electrophoresis and the 18SE pre-rRNA was detected by northern blotting using a probe hybridising to the 5ʹ end of ITS1.

The pre-ribosomal binding sites of the yeast homologues of the proteins studied here have been determined through structural studies and using a crosslinking and analysis of cDNA approach (CRAC [10,11];). The recently determined structures of cytoplasmic human pre-40S complexes suggests that the pre-ribosomal binding sites of these factors are largely conserved in metazoans [28,47]. However, little is known about whether these RNA elements alone are sufficient for the interaction with the proteins. We therefore tested the ability of our recombinant pre-40S factors to interact with *in vitro* transcribed fragments of rRNA/pre-rRNA. The regions of the 18S rRNA chosen included the 3ʹ major domain of 18S rRNA, where Nob1, Enp1 and Rio2 have been shown to bind in yeast (helices 33–40 (A) and helices 30–40 (B); Figure 5(A)), and the 3ʹ end of the 18S rRNA (helices 44, 45 (C and D); Figure 5(A)), which has been shown to be contacted by Dim1, Pno1 and Nob1 in yeast [10,11] and PNO1 and NOB1 in humans [28]. The key difference between the substrates C and D is the inclusion of sequence upstream of helix 44 in RNA C. We also used RNA substrates that encompassed both the 3ʹ end of 18S rRNA and the start of ITS1 (C+ ITS1 and D+ ITS1) that represent substrates for the final cleavage event by NOB1 at site 3. As the human ITS1 sequence is >85% GC rich, and therefore difficult to accurately amplify and transcribe, we used the mouse 18S/ITS1 sequence as the mouse ITS1 has normal GC levels. *In vitro* transcribed, [^32^P]-labelled RNAs were incubated with GST-tagged recombinant proteins and the resultant complexes were purified on glutathione sepharose. Co-precipitated RNAs were isolated, separated by denaturing polyacrylamide gel electrophoresis and detected using a phosphorimager. As a control, we also included GST-TIP48, a protein previously shown not to interact directly with RNA *in vitro* [48], which did not bind any of the RNAs tested here.

RIO2 and ENP1 did not bind to any of the RNA substrates, suggesting that their interaction with the pre-rRNA may require either additional regions of the rRNA or (an) additional protein(s). In contrast, we found that the 18S rRNA 3ʹ major domain RNAs (A and B) were bound by both NOB1 and PNO1, but not by DIM1 (Figure 5(B)). The RNA that encompassed both the 3ʹ end of 18S rRNA and the 5ʹ end of ITS1 (C+ ITS1) was bound by NOB1 and PNO1. Interestingly, this RNA was also weakly bound by DIM1, which is consistent with the presence of its methylation target within this sequence. Binding of NOB1, PNO1 and DIM1 to this rRNA region was significantly reduced, or abolished, in the absence of the ITS1 sequence (C), indicating that these proteins preferentially bind to the pre-rRNA rather than the mature rRNA as previously suggested for yeast Nob1 [44]. In addition, the removal of the sequence just upstream of helix 44, also reduced or abolished the binding of NOB1, PNO1 and DIM1 to this region (D and D+ ITS1). Recent work has demonstrated that the spacers between helix 28 and 44, and helix 44 and 45, base-pair in the late pre-40S complex (Supplementary Figure S6A) [28]. This structure, which is not present in the mature ribosome, is bound by PNO1 in the late pre-40S complexes. While our RNAs D and D+ ITS1 contain the elements needed to form this structure, we believe that the 3 Gs, added to the 5ʹ end for efficient transcription by T7 RNA polymerase, may interfere with the formation of the correct structure for helix 44 and/or 45 (Supplementary Figure S6A). This may explain why these two RNAs are not bound by NOB1, PNO1 and DIM1.

NOB1 and PNO1, two proteins that we have shown to stably interact, bind *in vitro* to the same two regions of the rRNA/pre-rRNA. It is therefore possible that the two proteins bind co-operatively to one or both of these RNAs. To test this, we compared the binding of GST-PNO1 and a pre-incubated mixture of the two proteins (His-NOB1 and GST-PNO1, immobilised on glutathione sepharose) to the upper 3ʹ domain of the 18S rRNA (RNA A) and the 3ʹ end of 18S rRNA and 5ʹ end of ITS1 (RNA C+ ITS1). We found that PNO1 binding to C+ ITS1 was not altered by the presence of NOB1 (Figure 5(C,D)), but in contrast, we observed a reproducible increase in binding of PNO1 to RNA A in the presence of NOB1. This indicates that the PNO1 and NOB1 complex has a higher affinity for the 3ʹ major domain of the 18S rRNA than PNO1 alone.

### Both the zinc ribbon and the PIN domain of human NOB1 are important for pre-rRNA processing

NOB1 and PNO1 bind two distinct regions of the 18S rRNA, namely the 3ʹ major domain (RNA A; H33-40) and the 3ʹ end of the 18S rRNA and the 5ʹ end of ITS1 (RNA C+ ITS1). In yeast, it has been suggested that H40 serves as a docking point for Nob1 and that the nuclease only associates transiently with the 3ʹ end of the 18S rRNA for cleavage of site 3 [10]. NOB1 has two potential RNA-binding domains: a PIN domain and a zinc-ribbon. Building on structural analysis of the archaeal NOB1 homologue, it was suggested that the PIN domain binds to the cleavage site while the zinc-ribbon binds to the H40 region of 18S rRNA [12]. To determine the importance of these two domains of human NOB1 for 18S rRNA maturation, HEK293 cell lines were generated for over-expression of full-length FLAG-NOB1 with a point mutation (D10N; FLAG-NOB1_PIN_; Figure 5(E) and Supplemental Figure S6B) in the catalytic PIN domain or full-length FLAG-NOB1 in which four evolutionarily conserved cysteines (C270, C273, C285 and C288) anticipated to mediate interactions between the zinc ribbon domain and RNA were converted to alanines (FLAG-NOB1_ZINC_) [12]. While over-expression of wild type FLAG-NOB1 in HEK293 cells did not significantly alter the levels of 18SE, or any other pre-rRNAs, over-expression of NOB1 with a mutated PIN domain (FLAG-NOB1_PIN_) had a dominant-negative defect on pre-rRNA processing by causing the accumulation of 18SE pre-rRNA (Figure 5(F)). In contrast, over-expression of NOB1 with a mutated zinc ribbon (FLAG-NOB1_ZINC_) had no effect on pre-rRNA processing.

It is possible that the integrity of the zinc ribbon domain is important for recruitment of NOB1 into the pre-ribosome. To test this, we performed immunoprecipitation experiments using extracts derived from cells expressing FLAG-tagged wild type or mutant NOB1 and analysed the co-purification of the 18SE pre-rRNA to determine whether the proteins are associated with pre-40S complexes. Both wild type protein (FLAG-NOB1) and NOB1 with an inactive PIN domain (FLAG-NOB1_PIN_) successfully co-purified 18SE pre-rRNA (Figure 5(G)). In contrast, the 18SE pre-rRNA did not co-purify with NOB1 containing an inactive zinc ribbon (FLAG-NOB1_ZINC_). This therefore indicates that the PIN domain of NOB1 is required for the conversion of the 18SE pre-rRNA to the mature 18S rRNA, while the zinc ribbon domain is required for the stable association of NOB1 with pre-40S complexes.

## Discussion

The discovery of differences between the ribosome assembly pathway in the yeast model system and humans, coupled with the emergence of numerous links between ribosome production and disease, highlight the need for detailed analyses of the factors involved in human ribosome assembly. A key aspect of pre-rRNA processing that differs between yeast and humans is the mechanism by which ITS1 is removed and the mature 3ʹ end of the 18S rRNA is formed. In this work, we have therefore analysed the involvement of a subset of protein factors in the late steps of human 18S rRNA maturation to identify similarities and differences between their functions in yeast and humans. Our data also provide new insight into possible mechanisms by which important events in late SSU biogenesis can be regulated and supports both a recent high resolution structure of human late pre-40S complexes [28] and a recent study on NOB1-PNO1 interactions [43].

The *N*^6^,*N*^6^-dimethylation of two adenosines close to the 3ʹ end of the SSU rRNA is conserved throughout evolution and is suggested to be important for translation fidelity [49]. In yeast, the methyltransferase Dim1 is recruited to nucleolar pre-ribosomes but modification of these sites takes place in the cytoplasm. We find that, as in yeast, human DIM1 is present in early pre-40S complexes but, in contrast, is largely absent from later pre-40S particles and is barely detectable in the cytoplasm [16,30]. Consistent with this, we observed that DIM1-mediated rRNA methylation begins on the 30S pre-rRNA and that the majority of nuclear 21S pre-rRNAs is methylated by DIM1. While we cannot exclude the possibility that a portion of DIM1 is co-exported within pre-40S complexes, these data are in line with an earlier observation that human DIM1 can act in the nucleus [16]. Furthermore, the recent structures of human cytoplasmic pre-40S complexes do not include DIM1 [28,47]. In yeast, it has been proposed that the cytoplasmic NTPase, Fap7, is involved in the release of Dim1 [50]. The human homologue of Fap7, hCINAP, is also involved in pre-40S maturation [27]. However, unlike Fap7, hCINAP is also present in the nucleus [51] and, if hCINAP is involved in the release of DIM1 from pre-ribosomes, this may explain why DIM1 acts and dissociates from pre-ribosomes in the nucleus in human cells. Despite the difference in the timing of DIM1-mediated methylation of the 18S rRNA sequence between yeast and humans, DIM1 is still essential for early 18S pre-rRNA processing steps in both systems. In human cells lack of DIM1 leads to turnover of early pre-ribosomal complexes. This supports the existence of a quality control pathway ensuring that only pre-ribosomal particles that contain DIM1 and can therefore be methylated, can undergo maturation [49].

Interestingly, our *in vitro* analyses revealed a robust interaction between DIM1 and the putative kinase RIO2. *Ct*Rio2 and archaeal Rio2 have previously been suggested to act as an ATPase rather than a kinase [45,46] but here, we identify DIM1 as a phosphorylation substrate of RIO2 *in vitro*, suggesting that human RIO2 can function as an active kinase during pre-40S maturation. It is possible that RIO2 could have two functions, perhaps acting as a kinase for DIM1, but also as an ATPase in the later cytoplasmic steps of pre-40S maturation. The relevance of the post-translational modification of DIM1 currently remains unknown. However, given the findings that DIM1 associates with very early pre-ribosomal complexes but that its methylation activity is not employed until later in the pre-40S biogenesis pathway, it is tempting to speculate that RIO2-mediated phosphorylation of DIM1 regulates the catalytic activity of DIM1. This is consistent with the observation that RIO2 is absent from early pre-ribosomes and is recruited to late nuclear pre-40S complexes. Alternatively, it is possible that phosphorylation of DIM1 by RIO2 is a metazoan-specific event that, perhaps together with hCINAP, causes release of DIM1 from pre-40S complexes, thereby explaining the difference in the timing of association of this methyltransferase with pre-40S complexes in yeast and humans.

The final maturation event in pre-40S biogenesis in both yeast and humans is cleavage of the 3ʹ end of the 18S rRNA by NOB1 [8,23,25,27]. Precise regulation of this step is therefore important to ensure the production of correctly assembled and processed 40S subunits. In yeast, various mechanisms for the regulation of Nob1-mediated cleavage of site D have been described. For example, it has been suggested that a conformational switch in the pre-18S rRNA induced by cleavage in ITS1 facilitates Nob1 access to its cleavage site [52], but this has been challenged in later studies [14]. Furthermore, the yeast RNA helicase Prp43 is proposed to catalyse structural rearrangements in the vicinity of site D to enable cleavage by Nob1 [8,13]. Interestingly, in human cells, PRP43 was not found in late pre-40S complexes and site 3 cleavage is not significantly affected by PRP43 depletion. This is supported by the finding that PRP43 is not present at significant levels in the cytoplasm [36]. This could suggest the involvement of an alternative RNA helicase during the final maturation of the 3ʹ end of 18S rRNA in humans. Furthermore, it could be that PRP43 function in human ribosome biogenesis is redundant. However, given the use of different pre-rRNA processing pathways to remove ITS1 in yeast and humans, it could also indicate that the pre-rRNA structure rearranged by Prp43 in yeast is not formed in metazoans.

Another mechanism by which the action of Nob1 is thought to be regulated is via its interactions with Pno1. The interaction between human NOB1 and PNO1 has recently been revealed [43] and supports the data we present here. In yeast, both proteins are recruited early in the nucleolus into the SSU processome and are predicted to remain bound until the final stages of 40S maturation in the cytoplasm. Based on the observation that yeast Pno1 dissociates from the pre-40S complex immediately prior to cleavage by Nob1, Pno1 was proposed to function as a chaperone, repressing the activity of Nob1 until the appropriate time for 3ʹ end cleavage of the 18S rRNA to occur [11]. Our data show that, similarly, human PNO1 is recruited to the early SSU processome and is required for intermediate pre-rRNA processing events in the nucleus. In contrast, we found that rather than binding early nucleolar complexes, NOB1 associates with later, nucleoplasmic pre-40S particles. Both NOB1 and PNO1 bind to two regions of the pre-rRNA *in vitro*, namely helices 34–40 and the 3ʹ end of the 18S rRNA. Interestingly, in the pre-40S complex, the structure of the junction between helix 44 and 45 is different to that seen in the mature 40S complex ([28] Supplementary Figure S6A). Our data support the importance of this structure in providing a binding site for NOB1, PNO1 and DIM1. Since NOB1 contains two RNA binding domains (zinc ribbon and PIN domain), it is likely that these pre-rRNA contacts are formed by different regions of the protein. We predict that the zinc ribbon domain, which is essential for the stable association of NOB1 with pre-40S complexes, likely binds to the 3ʹ major domain of the 18S rRNA, a point seen with the archaeal Nob1 [12], and also seen using RNA-protein crosslinking approaches [10,11]. Indeed, in the recent structure of the human pre-40S complex, in which NOB1 is bound to helix 45, the 3ʹ end of the 18S rRNA and ITS1, the zinc ribbon was not contacting the RNA [28]. Interestingly, we also find that PNO1 binds this same region and can also bind co-operatively with NOB1 to this site (Figure 5(C,D)). However, there are no other reports of an interaction between PNO1 and the 18S rRNA 3ʹ major domain in either the structural or crosslinking studies. A major structural transition is proposed to bring the catalytic PIN domain of NOB1/Nob1 into proximity of site 3/D [28,47,50,53] but this interaction is also potentially regulated by PNO1/Pno1. As both proteins were found to associate with the 3ʹ end of the 18S rRNA, this is in line with a model in which the binding of PNO1 to site 3 prevents access of NOB1 to its target sites ensuring that cleavage can only take place upon dissociation of PNO1 from pre-40S complexes [11,28].

Interestingly, our data on human NOB1 suggest an additional level of regulation of NOB1 activity during pre-40S biogenesis. Our data suggest multiple copies of NOB1 are present in pre-40S complexes. This model is supported by the higher levels of NOB1 compared to other pre-40S components (e.g. PNO1, DIM1, RIO2; Figure 2(A)) and the detection of human NOB1 multimerisation both *in vitro* (Figure 4(A)) and *in vivo* (Figure 2(C,D)). Interestingly, multimerisation of other PIN domain proteins has been suggested to be important for their catalytic activity and *in vitro* dimerisation of yeast Nob1 has been previously reported [42]. Furthermore, it has been proposed that recombinant yeast Nob1 binds as a tetramer to pre-18S rRNA fragments that contain cleavage site D (site 3 in humans) [44]. This raises the possibility that the recruitment of additional molecule(s) of NOB1 to pre-40S complexes contributes to the regulation of site 3 cleavage. While the currently available cryo-EM structures of eukaryotic pre-40S complexes containing NOB1 and PNO1 all possess a single copy of NOB1, it is possible that NOB1 multimerisation occurs on later pre-40S particles, following the dissociation of PNO1.

Taken together, our results support a dynamic model in which the sequential recruitment, activation and release of numerous factors enable efficient and accurate maturation of the 3ʹ end of the 18S rRNA. Although dimethylation of 18S-A1850/1 and site 3 cleavage are mediated by the same enzymes in humans as in yeast, temporal differences in the recruitment and action of these enzymes in the two species have emerged. While the relevance of these differences remains to be elucidated, events such as phosphorylation of DIM1 by RIO2, and NOB1 post-translational modification and multimerisation, likely represent key mechanisms by which these steps can be regulated.

## Materials and methods

### Generation of stable cell lines, cell culture and RNAi

HEK293 Flp-In T-Rex (Invitrogen/Thermo Fisher) and HeLa cells were grown at 37°C with 5% CO_2_ in DMEM supplemented with 10% foetal calf serum (FCS), according to standard protocols. To generate cells stably expressing the proteins of interest under the control of a tetracycline-inducible promoter, the coding sequences of NOB1 (NM_014062.2), PNO1 (NM_020143.3), DIM1 (NM_014473.3) and RIO2 (NM_018343.2) were first cloned into a pcDNA5 vector for expression of proteins with an N-terminal 2xFlag-PreScission protease site-His_6_ (Flag) tag. Site-directed mutagenesis was used to generate constructs for expression of catalytically inactive NOB1 (D10N; NOB1_PIN_) or NOB1 with mutations in the zinc ribbon domain (C270A, C273A, C285A and C288A; NOB1_ZINC_). These plasmids, or the empty pcDNA5 plasmid, were transfected into Flp-In T-Rex HEK293 cells and cells that had integrated the plasmid into their genome were selected according to the manufacturer’s instructions. Expression of tagged proteins was induced by addition of 1–1000 ng/ml tetracycline for 24 h before harvesting, as appropriate. For RNAi-mediated knockdowns, cells were transfected with siRNA duplexes (50 nM; Supplementary Table S1) using Lipofectamine RNAiMAX reagent (Invitrogen/Thermo Fisher) according to the manufacturer’s instructions and harvested after 60 h. To block ribosome biogenesis 0.1 μg/μl actinomycin D (ActD) was added for 18 h.

### RNA analysis (northern blotting and S1 nuclease mapping)

RNA was extracted from HEK293 and HeLa cells using TRI regent (Sigma-Aldrich) according to the manufacturer’s instructions. For northern blotting, 3 μg of total RNA was separated by electrophoresis on a 1.2% agarose-glyoxal gel (RNAs >300 nt) or a denaturing (7 M urea) 10% polyacrylamide gel (RNAs <300 nt) and transferred to a nylon membrane. Probes used were 5ʹ-[^32^P]-labelled DNA oligonucleotides hybridising to the 5ʹ end of ITS1 and between sites 2a and 2 in ITS1, as described previously [23]. Random prime [^32^P]-labelled DNA probes for the detection of the U3 snoRNA, the U1 snRNA and ETS3-containing pre-rRNAs, were prepared as described in [54]. RNAs were detected using a phosphorimager and signals were quantified using ImageQuant software. S1 nuclease mapping was performed as previously described [41] using a 5ʹ-[^32^P]-labelled probe hybridising across the A’ cleavage site. Cleaved and uncleaved RNA fragments were separated by denaturing polyacrylamide gel electrophoresis and detected using a phosphorimager.

### rRNA modification analysis by RNase H cleavage

Analysis of the extent of *N*^6^,*N*^6^-dimethylation of 18S-A1850 and 18S-A1851 using an RNase H-based cleavage assay was essentially performed as previously described [55]. In brief, 4 μg of total RNA harvested from siRNA-treated cells was annealed to a chimeric RNA/DNA oligonucleotide (5ʹ-mAmGmGdTdTdCdAmCmCmUmAmCmGmGmAmAmAmC-3ʹ, where mN is 2ʹ-*O*-methylated RNA nucleotides and dN are DNA nucleotides) that anneals across the target modification site. RNA-chimera complexes were then treated with *E. coli* RNase H for 30 min at 37°C to cleave RNA-DNA hybrids or left untreated. Reactions were stopped by addition of EDTA to a concentration of 0.2 mM and RNAs were extracted using phenol:chloroform:isoamylalcohol, ethanol precipitated and analysed by northern blotting. Quantitation of bands was performed using Image Quant software.

### Glycerol gradients and immunoprecipitation

Whole cell extracts were prepared from HEK293 cells using sonication as previously described [56]. For immunoprecipitation, extracts were incubated with anti-Flag-coupled Protein G sepharose and co-precipitated RNAs and proteins were extracted and analysed by northern or western blotting (antibodies listed in Supplementary Tables S2). Whole cell extracts were separated on 10–40% glycerol gradients by centrifugation at 52,000 rpm in an SW 60 Ti rotor for 90 min. After fractionation of the gradients, proteins were analysed by western blotting or alternatively, RNAs were isolated and analysed by northern blotting.

### Recombinant protein expression

The coding sequences of NOB1, PNO1, DIM1, RIO2 and ENP1 (NM_004053.3) were cloned into either the pGEX-6P-1 vector for expression of proteins with an N-terminal GST tag or the pET200a vector for expression of proteins with an N-terminal His_6_ tag. Site-directed mutagenesis was used to introduce point mutations (K123A and D246A) within the catalytic site of RIO2 (RIO2_KD_). Recombinant proteins were expressed in *E. coli* BL21 Codon Plus cells and cells were lysed in a buffer containing 50 mM Tris-HCl pH 8.0, 10% glycerol, 300 mM NaCl, 5 mM MgCl_2_ and 0.1% Tween20. The lysate was then incubated with either glutathione sepharose (GST-tagged proteins) or Ni-NTA resin (His-tagged proteins). After washing steps, GST-tagged proteins were eluted in lysis buffer supplemented with 50 mM glutathione pH 8.0. His-tagged proteins were eluted using a 50–500 mM imidazole gradient. All proteins were desalted using HiTrap Desalting columns (GE Healthcare) prior to use. To remove the GST-tag from purified proteins, 100 μg of GST-tagged protein was incubated with ~1 μg GST-tagged PreScission protease for 1 h at 4°C. The protease and cleaved tag were then removed using glutathione sepharose beads.

### In vitro analysis of protein-protein and RNA-protein interactions, and kinase assays

*In vitro* binding assays were performed as previously described [57]. For protein-protein interactions, 50 ng of GST-tagged bait protein was incubated with equal amounts of His-tagged prey protein in a buffer containing 50 mM Tris-HCl pH 8.0, 10% glycerol, 300 mM NaCl, 5 mM MgCl_2_ and 0.1% Tween20 for 1 h at 4°C. GST-tagged proteins or complexes were isolated using glutathione sepharose, separated by SDS-PAGE and analysed by western blotting. For analysis of RNA-protein interactions, RNA substrates corresponding to fragments of the mouse 18S rRNA and ITS1 were transcribed from PCR products, containing the T7 promoter sequence, using T7 RNA polymerase in the presence of [^32^P]-α-UTP. GST-tagged bait proteins, or pre-formed complexes of GST-tagged and His-tagged proteins, were immobilised on glutathione sepharose before addition of radiolabelled transcripts. Co-purifying RNAs were then isolated, separated by denaturing polyacrylamide gel electrophoresis and visualised using a phosphorimager. For *in vitro* kinase assays, 10 ng of recombinant GST, GST-RIO2 or GST-RIO2_KD_ was incubated with an equivalent amount of putative substrate proteins in a buffer containing 20 mM Tris-HCl pH 8.0, 300 mM KCl, 5 mM MgCl_2_, 0.1% Tween20, 10% glycerol. 1 μCi of [^32^P]-γ-ATP, either alone or with 0.01 or 0.001 mM cold ATP were added and reactions were incubated at 37°C for 30 min. The reactions were stopped by addition of SDS-loading dye, samples were separated by SDS-PAGE and proteins were visualised by Coomassie staining or using a phosphorimager.
