# Supplementary material for: Interactions and activities of factors involved in the late stages of human 18S rRNA maturation
Source: RNA Biol. 2019 Jan 13;16(2):196–210. doi: 10.1080/15476286.2018.1564467 (PMC6380343; doi:10.1080/15476286.2018.1564467)
Supplement: Supplemental Material [file krnb-16-02-1564467-s001.pdf]

Supplementary material

Interactions and activity of factors involved in the late stages of human 18S rRNA maturation

Katherine E. Sloan, Andrew A. Knox, Graeme R. Wells, Claudia Schneider and Nicholas J. Watkins

Supplementary Figures

Supplementary Figure S1. RNAi-mediated depletion of selected 18S rRNA maturation factors. A) HeLa cells were transfected with control siRNAs or those targeting the indicated 18S rRNA biogenesis factors and, after 60 h, cells were harvested. The levels of each protein were determined using antibodies against the endogenous proteins and the cytoskeletal protein actin or the box C/D snoRNP component fibrillarin (FIB) were used as loading controls. Antibody used is indicated to the right of each panel. **B)** For DIM1, where we lacked an antibody that recognises the endogenous protein, control siRNAs or those targeting DIM1 were transfected in the HEK293 cells expressing FLAG-tagged DIM1 and the levels of DIM1, relative to actin, were determined by western blotting using an anti-FLAG antibody.

Supplementary Figure S2. Northern blot mapping of aberrant pre-rRNA fragments detected upon depletion of DIM1. A) Schematic view of the 47S, 41S and 21S pre-rRNA transcripts (black) and the aberrant 16S* intermediate (grey). Mature rRNA sequences are shown as black boxes and internal and external transcribed spacers are shown as black lines (ITS - internal transcribed spacer; ETS – external transcribed spacer). Pre-rRNA cleavage sites are indicated on the 47S pre-rRNA transcript by vertical lines. The relative hybridisation positions of probes used in northern blotting are indicated. **B)** HeLa cells were transfected with control siRNAs or siRNAs against DIM1 and after 60 h RNA was extracted and separated on an agarose-glyoxal gel. The RNAs were then transferred to a nylon membrane and stained with methylene blue (MB); the mature rRNAs detected served as a loading control. The membranes were then probed with various probes (indicated below each panel; see panel A) and then images were captured using a phosphorimager and quantified using Image Quant software. The identity of the rRNAs and pre-rRNAs are indicated to the right of each panel.

Supplementary Figure S3. Expression of FLAG-DIM1 to endogenous U3-55K levels. HEK293 cells for expression of FLAG-tagged U3-55K, PNO1, DIM1 or NOB1 were treated with tetracycline to induce expression of the FLAG-tagged proteins to endogenous levels (U3-55K, PNO1, NOB1; see also Figure 2A) or to the same level as U3-55K (DIM1). Cells were harvested and proteins were analysed by SDS-PAGE followed by western blotting using anti-FLAG (upper panel) and anti-U3-55K (lower panel) antibodies.

Supplementary Figure S4. RNase H-based assay for the detection of *N*^{6},*N*^{6} dimethylation of adenosines 1850 and 1851 of the 18S rRNA. Schematic view of the pre-rRNA intermediates detectable by northern blotting using a probe hybridising to the 5' end of ITS1 upon RNase H cleavage directed to 18S-A1850/1. The major precursors of the 18S rRNA are shown schematically in black and the cleaved products are shown in grey. The sizes of all these pre-rRNA species are given in nucleotides (nt) to the right of the panel.

Supplementary Figure S5. RIO2 phosphorylates itself and DIM1 but not NOB1, PNO1 or ENP1 *in vitro*. A) GST-RIO2 was incubated with recombinant proteins indicated above the panel in the presence of $\gamma[^{32}P]$-labelled ATP and 0.001mM ATP. Proteins were separated by SDS-PAGE and labelled proteins were detected using a phosphorimager. Protein inputs were run alongside the reactions and proteins were detected by Coomassie staining. and a non-specific phosphorylated protein is indicated by an asterisk. **B)** GST-RIO2 or GST-RIO2$_{KD}$, were incubated with His-tagged DIM1 in the presence of $\gamma[^{32}P]$-labelled ATP and 0.001mM ATP. Proteins were separated by SDS-PAGE and labelled proteins were detected using a phosphorimager. The bands corresponding to the input proteins are marked on the right. The data were quantified, comparing the relative levels of phosphorylated protein for RIO2 and DIM1 between the RIO2 and RIO2$_{KD}$ lanes and plotted as mean ± standard error of these two experiments and the equivalent reaction in Figure 5C. A t-test was performed comparing phosphorylation of the proteins in RIO2 and RIO2$_{KD}$ reactions. ** $p < 0.01$, *** $p < 0.001$.

Supplementary Figure S6. Secondary structures of the NOB1 and PNO1 RNA-binding substrates and the expression of wild type FLAG-NOB1 and FLAG-NOB1 mutants. A) Secondary structure of the 3' major and minor domains of the 18S rRNA is shown with the positions of fragments used for protein-RNA binding analysis highlighted in colours. **B)** HEK293 cells for expression of FLAG-tagged NOB1, or NOB1 mutants (indicated at the top) were treated with tetracycline (Tet) to induce expression of the FLAG-tagged proteins. Cells were harvested and proteins were analysed by SDS-PAGE followed by western blotting using anti-FLAG and anti-Karyopherin (loading control) antibodies.

Supplementary Tables

Supplementary Table S1. siRNAs used in this study.

siRNA	Sequence (5'-3')	Source/reference
Control	CGTACGCGGAATACTTCGAdTdT	(GL2) MWG (1)
DIM1	GAUUCAACGCAGAAGGUAUUU	Dharmacon Smartpool
	GAUGGUCUAGUAAGGAUAAUU	
	CUUAAGACCAACUGAUGUAUU	
	ACCAGAAGAUUUCAGCAUAUU	
NOB1	UCACCGAGGAUCAGCGCUUUU	Dharmacon Smartpool (2)
	GCAAAUUGGAUGGGCGUCUUU	
	ACAGAAAAGAUGACAGCGAUU	
	AGAAAGUGUCCGUGACCGUUU	
PNO1	GCUAACAGAUACACACCAUUU	Dharmacon Smartpool
	GGACUUCAGAUACGCUUUAUU	
	AGAGAAUGUGACACGGACAUU	
	CUUAAAAGGGCUCCGAACAUU	
PRP43	UUGAAAGUAGUUCUAUAUAdTdT	MWG
	CATTCTGATTTCATAAATTAAdTdT	
ENP1	CGAAAUCAGGCGUGAGCUUdTdT	MWG (3)
	AGAUGUUCAUGAACAAGAAdTdT	

Supplementary Table S2. Antibodies used in this study.

Antibody	Source
NOB1	Eurogentec (this study)
PNO1	Eurogentec (this study)
Fibrillarin	Santa Cruz (sc25397)
RIO2	Santa Cruz (sc136837)
GST	Santa Cruz (sc138)
His	Santa Cruz (sc8036)
PRP43	Bethyl (A300-390A)
Flag	Sigma-Aldrich (F1804)
Flag	Sigma-Aldrich (F7425)
Karyopherin	Santa Cruz (sc11367)
hU3-55K	(4)

Supplementary references

1. Elbashir, S.M., Harborth, J., Lendeckel, W., Yalcin, A., Weber, K. and Tuschl, T. (2001) Duplexes of 21-nucleotide RNAs mediate RNA interference in cultured mammalian cells. *Nature*, **411**, 494-498.
2. Sloan, K.E., Mattijssen, S., Lebaron, S., Tollervey, D., Pruijn, G.J. and Watkins, N.J. (2013) Both endonucleolytic and exonucleolytic cleavage mediate ITS1 removal during human ribosomal RNA processing. *J Cell Biol*, **200**, 577-588.
3. Carron, C., O'Donohue, M.F., Choesmel, V., Faubladier, M. and Gleizes, P.E. (2011) Analysis of two human pre-ribosomal factors, bystin and hTsr1, highlights differences in evolution of ribosome biogenesis between yeast and mammals. *Nucleic Acids Res*, **39**, 280-291.
4. Granneman, S., Pruijn, G.J., Horstman, W., van Venrooij, W.J., Luhrmann, R. and Watkins, N.J. (2002) The hU3-55K protein requires 15.5K binding to the box B/C motif as well as flanking RNA elements for its association with the U3 small nucleolar RNA in Vitro. *J Biol Chem*, **277**, 48490-48500.

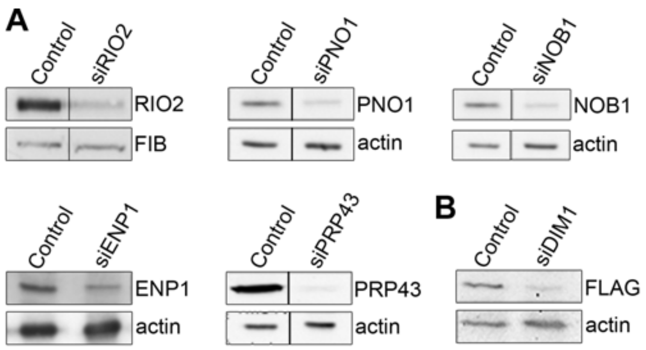

Supplementary Figure S1

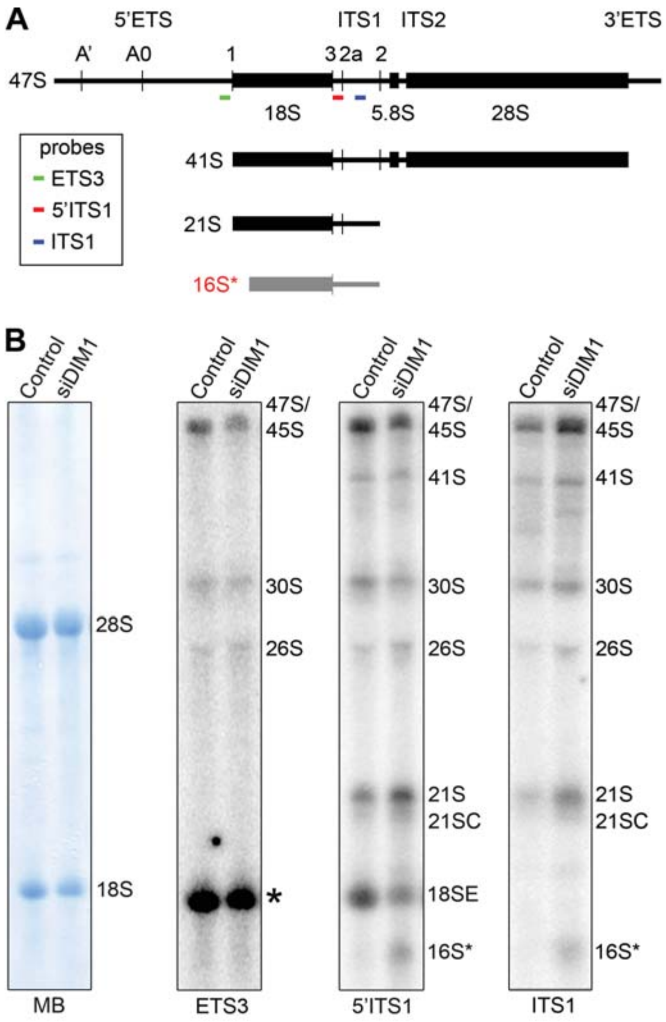

Supplementary Figure S2

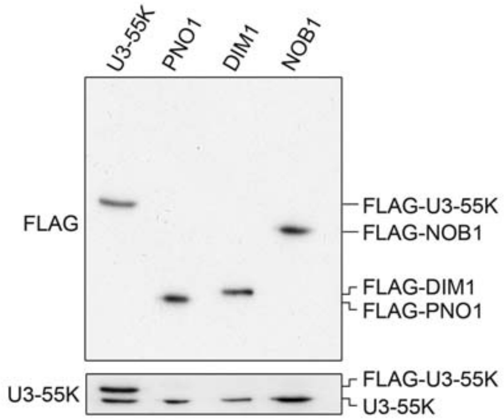

Supplementary Figure S3

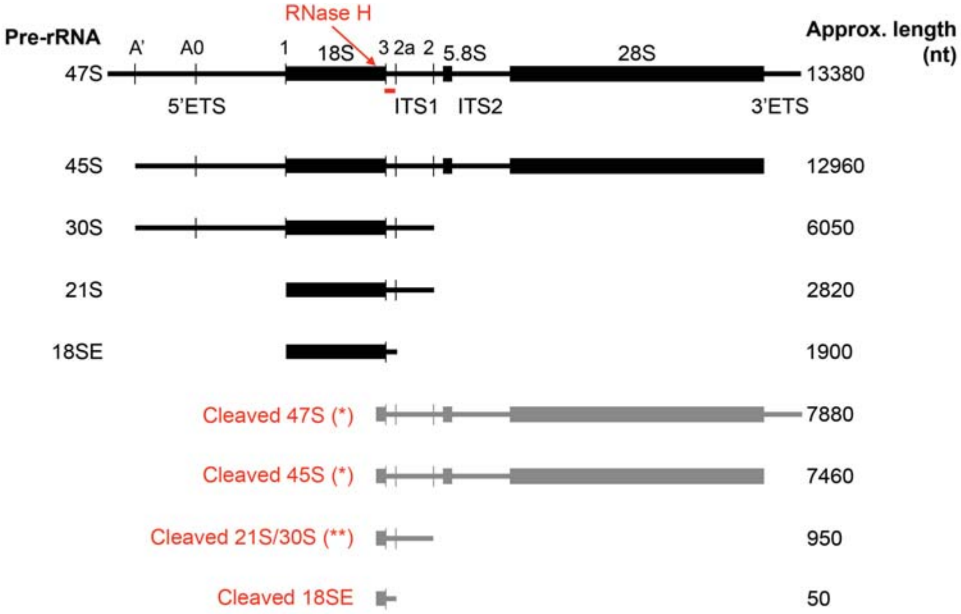

Supplementary Figure S4

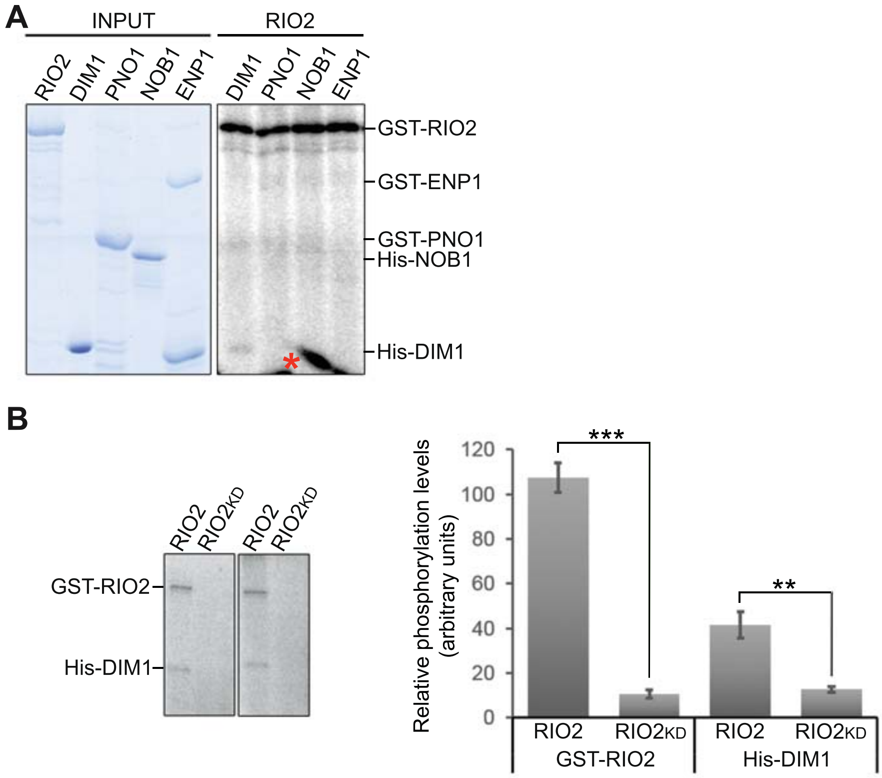

Supplementary Figure S5

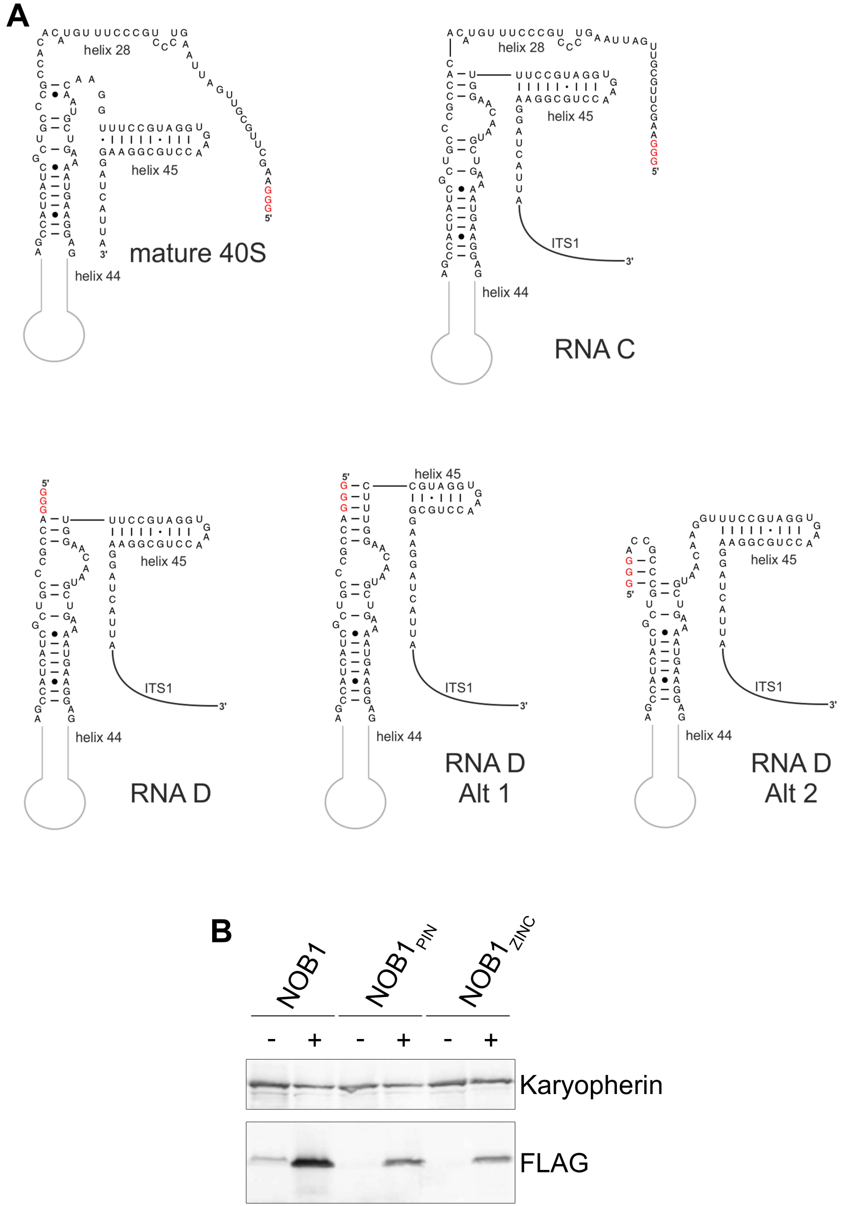

Supplementary Figure S6
